# Interactive Conversational Agents for Perinatal Health: A Mixed Methods Systematic Review

**DOI:** 10.3390/healthcare13040363

**Published:** 2025-02-08

**Authors:** Samira Amil, Sié-Mathieu-Aymar-Romaric Da, James Plaisimond, Geneviève Roch, Maxime Sasseville, Frédéric Bergeron, Marie-Pierre Gagnon

**Affiliations:** 1 Centre NUTRISS, Institut sur la Nutrition et les Aliments Fonctionnels (INAF), Québec, QC G1V 0A6, Canada; samira.amil.1@ulaval.ca; 2 VITAM-Centre de Recherche en Santé Durable, Québec, QC G1V 0A6, Canada; sie-mathieu-aymar-romaric.da.1@ulaval.ca (S.-M.-A.-R.D.); james.plaisimond.1@ulaval.ca (J.P.); genevieve.roch@fsi.ulaval.ca (G.R.); maxime.sasseville@fsi.ulaval.ca (M.S.); 3 Faculté des Sciences Infirmières, Université Laval, Québec, QC G1V 0A6, Canada; 4 Centre de Recherche du CHU de Québec, Université Laval, Québec, QC G1E 6W2, Canada; 5 Centre de Recherche du CISSS de Chaudière-Appalaches, Lévis, QC G6V 3Z1, Canada; 6 Bibliothèque-Direction des Services-Conseil, Université Laval, Québec, QC G1V 0A6, Canada; frederic.bergeron@bibl.ulaval.ca

**Keywords:** conversational agents, chatbots, systematic review, maternal health care, perinatal health care

## Abstract

**Background:** Interactive conversational agents (chatbots) simulate human conversation using natural language processing and artificial intelligence. They enable dynamic interactions and are used in various fields, including education and healthcare. **Objective:** This systematic review aims to identify and synthesize studies on chatbots for women and expectant parents in the preconception, pregnancy, and postnatal period through 12 months postpartum. **Methods:** We searched in six electronic bibliographic databases (MEDLINE (Ovid), CINAHL (EBSCO), Embase, Web of Science, Inspec, and IEEE Xplore) using a pre-defined search strategy. We included sources if they focused on women in the preconception period, pregnant women and their partners, mothers, and fathers/coparents of babies up to 12 months old. Two reviewers independently screened studies and all disagreements were resolved by a third reviewer. Two reviewers independently extracted and validated data from the included studies into a standardized form and conducted quality appraisal. **Results:** Twelve studies met the inclusion criteria. Seven were from the USA, with others from Brazil, South Korea, Singapore, and Japan. The studies reported high user satisfaction, improved health intentions and behaviors, increased knowledge, and better prevention of preconception risks. Chatbots also facilitated access to health information and interactions with health professionals. **Conclusion:** We provide an overview of interactive conversational agents used in the perinatal period and their applications. Digital interventions using interactive conversational agents have a positive impact on knowledge, behaviors, attitudes, and the use of health services. Interventions using interactive conversational agents may be more effective than those using methods such as individual or group face-to-face delivery.

## 1. Introduction

For many women, gender-related social and physiological inequalities significantly impede the attainment of an overall state of health and, by extension, their ability to achieve their full potential as individuals. Women’s health is thus a global priority [1]. Indeed, although women have a higher life expectancy (74.2 years) than men (69.8 years), they have a higher morbidity rate than men and have greater recourse to health and social services, particularly for their sexual, reproductive, and mental health needs throughout the preconception and perinatal periods [2].

The “preconception period” is most often defined as the three months prior to conception [3,4]. The “perinatal period” is defined as from conception to one year postpartum [5]. These life stages involve changes at the biological, psychological, social, and cognitive levels that could negatively impact the health of women and babies before, during, and after birth (perinatal health). It is thus essential to consider women’s needs specific to these life stages in the provision of care and services [6].

To meet these needs, women are increasingly turning to websites, social media, and smartphone apps for information [7]. The internet remains the most widely used tool for finding information on perinatal topics [8], and is also used as a virtual space for sharing experiences and peer support [9].

Recent studies have begun to explore the landscape of chatbot technology in maternal health. For example, Kaneho et al. [10] conducted a survey of existing chatbots specifically designed for maternal healthcare, providing valuable insights into current offerings and their potential impact on perinatal health. The use of technology was amplified during the COVID-19 pandemic, when most households worldwide faced restrictions and social isolation, limiting expectant and new parents’ access to help and support [11].

In recent years, considerable technological advances have allowed for the development of conversational agents (chatbots) capable of interacting with a human using artificial intelligence. Chatbots are software packages that interact with users through text or voice exchanges and generate speech through natural language processing [10,12,13]. With the development of increasingly powerful and connected devices, smartphone chatbots are now widely used by consumers for everyday tasks such as information retrieval [14].

Considering their growing capabilities, chatbots have the potential to play an increasingly key role in the health care field, assisting women and expectant parents in the perinatal period and making the pregnancy and childbirth experience positive, allowing women and their babies to reach their full potential for health and well-being [14,15,16,17]. Indeed, the perinatal period, from conception to one year postpartum, is a crucial time when future parents and parents need health-care follow-up and information on many topics, including maternal-fetal needs, the course of pregnancy and its complications, vitamin and mineral supplementation, delivery and associated risks, postpartum, postpartum contraception, breastfeeding, baby’s diet and dietary diversification, psychomotor development of the baby, vaccination, psychological and social adjustment to parenthood, and infant care abilities. They usually turn to electronic sources to find answers to their questions, but the information found there is not always credible or is even contradictory [15,17]. It is thus important to explore this question: What is the effectiveness and acceptability of interactive conversational agents in supporting various aspects of perinatal health for expectant and recent parents?

To our knowledge, no systematic review of chatbots in perinatal health has been undertaken. Our objective was to systematically identify sources and synthesize the evidence on chatbot interventions to support women and expectant parents in preconceptions, pregnancy, and postpartum through 12 months postpartum.

To address our research question, we conducted a systematic review following the PRISMA guidelines. A comprehensive search strategy was applied across six databases, including studies published between 2000 and 2022 in multiple languages. Eligible studies evaluated the effectiveness and acceptability of conversational tools in perinatal health, based on pre-defined PICOS criteria.

The paper is organized as follows: the Materials and Methods section describes the study design, inclusion criteria, and data synthesis. The Results section presents the findings, supported by four key tables summarizing study characteristics (Table 1), primary outcomes (Table 2), secondary outcomes (Table 3), and quality ratings (Table 4). The Discussion section analyses the implications of these findings and highlights research gaps, while the Conclusion section highlights the contribution of the review to advancing digital health interventions in perinatal care.

## 2. Materials and Methods

### 2.1. Overview

We used the Preferred Reporting Items for Systematic Reviews and Meta-Analysis (PRISMA) guidelines to plan this review [30]. The protocol of this review was registered on the International Prospective Register of Systematic Reviews (PROSPERO) (CRD42023376991).

### 2.2. Eligibility Criteria

We followed the PICOS (Population, Intervention, Comparison, Outcome and Study) framework to design the eligibility criteria.

Population: We included all trials that involved women in the preconception period, pregnant women and their partners, and mothers and fathers/co-parents of babies up to 12 months of age.

Interventions: We included any intervention with a conversational agent that allows participants to interact bidirectionally, either by voice or chat, or a combination of both. We excluded any intervention with a conversational agent used only to collect information or developed for the training of health professionals or students.

Comparator: We considered all comparators, including no intervention, usual care, or any other type of intervention.

Outcomes: We considered all outcomes reported in the studies that are related to the effectiveness of interactive conversational agents in any area of perinatal health or well-being such as pregnancy-related information seeking, childbirth, breastfeeding, dietary diversification, health, and support resources.

We were also interested in the non-clinical metrics such as user engagement, duration of adherence and duration of individual interaction, user experience, and acceptability.

Setting: We included studies taking place in primary health care, hospitals, community settings, third-sector organizations, or any other setting.

All types of studies were included (qualitative, quantitative, and mixed methods) if they had health-related outcomes.

### 2.3. Search Strategy

In collaboration with a librarian (FB), we developed a search strategy in six electronic bibliographic databases (MEDLINE (Ovid), CINAHL (EBSCO), Embase, Web of Science, Inspec, and IEEE Xplore). The sensitivity of the search strategy was tested before starting the screening process with five key articles.

Search strings combined free terms and, when supported, controlled vocabulary. The reference lists of relevant articles were also screened to ensure that all eligible studies were captured. All studies published from January 2000 to July 2022 in English, French, Spanish, Portuguese, and Italian (languages understood by the reviewers) were considered regardless of the study design.

We excluded the following types of publications: editorial comment, opinion, informative review, conference abstract, commentary, systematic review, and protocol. Grey literature such as dissertations, theses, and conference proceedings were not included. (The search strategy is presented in Appendix A).

### 2.4. Data Collection

We used the online platform Covidence to conduct the review (Covidence systematic review software). We imported all references to the tool and most duplicates were automatically removed. Two reviewers independently assessed the title and abstract of each reference using the criteria. We then obtained the full text of included references, and two reviewers independently assessed the studies for final inclusion. Two reviewers appraised the quality the studies included with the Mixed Methods Appraisal Tool (MMAT) [31]. Any disagreement was resolved by a third reviewer.

### 2.5. Data Extraction

An extraction grid was used in Covidence for the abstraction of data from included studies. The following data were collected for each study: first author, year of publication, type of study, type of technology (chat, voice, or a combination of both), intervention components and characteristics, study duration (if applicable), participants and setting characteristics, and health outcomes and non-clinical outcomes (if applicable). Two reviewers extracted data independently, and in case of missing data, direct requests were made to the study authors to supply the information.

### 2.6. Data Synthesis

We conducted descriptive and thematic analyses and presented the results in the form of a structured narrative synthesis of the main technologies used in perinatal care by fields of application. We used a prespecified thematic analysis grid based on 3 main categories of outcomes: 1, Antecedents; 2, Healthy behaviors; 3, Health status or health services utilization. We also conducted a narrative synthesis of qualitative findings, and we summarized the strengths and weaknesses reported for each conversational agent.

## 3. Results

**Figure 1:**
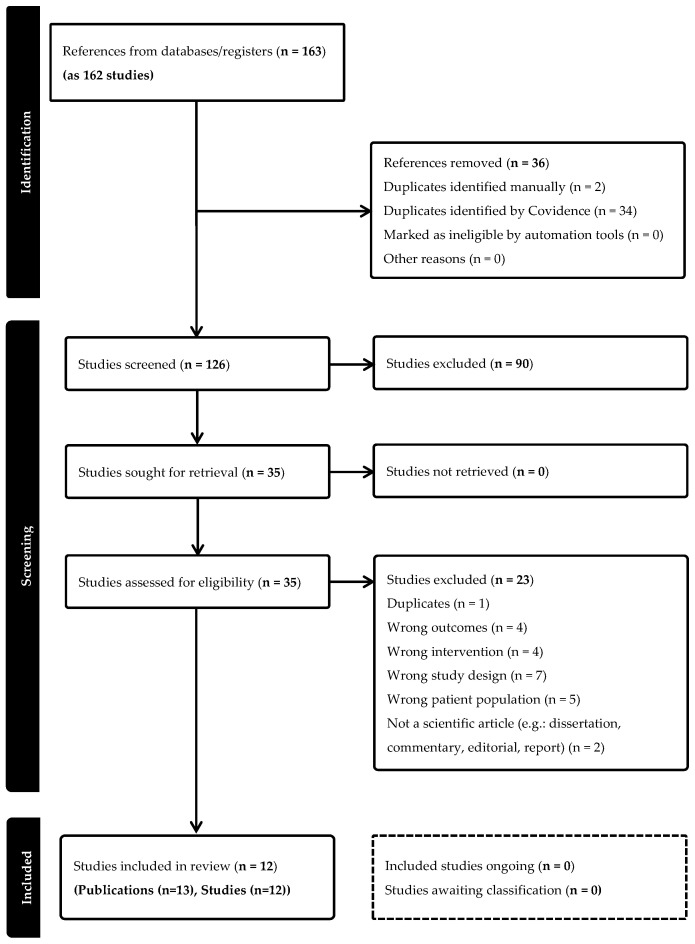
PRISMA flow diagram for inclusion of reviews.

A total of 162 publications were retrieved, and 36 duplicates were removed manually. The remaining 126 publications were screened by independent reviewers using titles and abstracts. Thirty-six publications were screened in full text, among which thirteen were retained. Two publications related to the same study were considered jointly, resulting in twelve studies suitable for inclusion in this review (see Figure 1).

A PRISMA flowchart describes the identification of studies, the screening process, and the application of inclusion and exclusion criteria [32] (Figure 1).

The selected studies were conducted between 2013 and 2022 (see Table 1). Seven studies were conducted in the USA, two in Brazil, one in the Republic of Korea, one in Singapore, and one in Japan. These studies evaluated the use and effectiveness of chatbots in various aspects of perinatal health. Most studies focused on the preconception period, particularly fertility [27], preconception health [27,28], and preconceptional-related risks [19,24,25,26]. Other studies focused on childbirth, specifically the mode of birth after cesarean [20], stress [29], and parental mental health during the perinatal period [21]. Some studies focused on sleep and neonatal dietary diversification [29], and various breastfeeding-related behaviors, including intentions, attitudes, and self-efficacy [22]. Only one study focused on child health promotion [18], and another on lifestyle changes in women of childbearing age [23].

The study population was generally female and ranged in age from 18 to 50 years. Only two studies included men aged 38 to 40 years [21] or parents aged 21 years and older [29]. Interventions to promote breastfeeding, dietary diversification, and infant health were mainly aimed at young primiparous women (new mothers) or young parents. In the trials that focused on the mode of birth after cesarean, the authors included women who already had a cesarean. The number of participants recruited ranged from 15 to 927 participants. All the chatbots studied interacted with their users via text messages, four via text messages and voice, three via audio, and seven integrated avatars into their chatbots.

Of the 12 studies included in our systematic review, 50% were randomized clinical trials (6/12), 25% were mixed methods studies (3/12), 17% (2/12) were observational descriptive studies (two or more phases), and only one study (0.9%) had a qualitative research design.

### 3.1. Primary Outcomes Measured

The present review brings together heterogeneous studies with specific objectives and therefore different but complementary results, as shown in Table 2, which summarizes the main findings by topic.

Of the twelve included studies, six [18,19,21,23,28,29] assessed chatbot usability; three [23,25,26] preconception risks defined as health and nutrition risk factors that can have an impact on maternal and child health before conception; two studies [23,27] assessed knowledge; and one [22] breastfeeding. To assess usability, Barreto et al. [18] measured newborn mothers’ user experience and satisfaction with the chatbot. The results indicate that women’s level of agreement with the simplicity, good quality of information, clarity of content, usefulness, and overall satisfaction with the chatbot, was over 90%. In Wong’s study [29], the usability assessment showed that parents found the chatbot easy to use (mean = 4.08, SD = 0.74; 1 = very difficult, 5 = very easy) and that they were satisfied (mean = 3.81, SD = 0.90; 1 = very dissatisfied, 5 = very satisfied).

Bickmore et al. [19] evaluated the acceptance, usability, and use of a chatbot to screen women for preconception care risks and treat them via an animated web-based virtual health advisor. Differences between younger (18–25 years old) and older (26–34 years old) in relation to chatbot acceptance and utilization were explored. No significant differences were found between the two age groups for either of these parameters. Chung et al. [21] evaluated a chatbot based on a question-and-answer knowledge database for obstetric and mental health care for perinatal women and their partners. The results indicate that, apart from ease of learning, total usability and the other three sub-factors (usefulness, ease of use, and satisfaction) had significant positive associations with each other.

In Montenegro’s study [28], the usability assessment showed that the most significant and positive construct was related to chatbot performance expectations (mean = 4.61, SD = 0.74), while the construct with the least positive influence on pregnant women was facilitating conditions (mean = 3.30, SD = 1.24). From these results, it emerges that pregnant women believe that interaction with the chatbot has educated them and that their physician would approve their use [28].

Edwards et al. [22] looked at the evaluation of an animated, interactive computer agent designed to provide breastfeeding information and support mothers interested in breastfeeding. The results show that mothers who used the chatbot were more likely to breastfeed exclusively after being exposed to the chatbot (*p* < 0.05) [22].

Gardiner et al. [23] assessed the feasibility of using a chatbot to teach lifestyle modifications to urban women of childbearing age. In this regard, the results showed that after one month, among women randomized to the chatbot, alcohol consumption to reduce stress significantly decreased (*p* = 0.03) and daily fruit consumption increased by an average of two servings compared with the control (*p* = 0.04) [23]. Regarding knowledge assessment, this study compared food knowledge before and after the intervention, and the results were not statistically significant between the two study groups [23].

Maeda et al. [27] assessed the impact of a chatbot on fertility knowledge and the results indicate that fertility knowledge improved over time in the intervention group (chatbot) (+9.1 points, *p* < 0.001), control group 1 (+14.9 points, *p* < 0.001), and control group 2 (+1.1 points, *p* = 0.24). Preconception risk assessment was carried out in the studies by Gardiner et al. [24], and Jack et al. [25,26]. The results showed that the use of a chatbot for preconception risk assessment had a statistically significant positive effect between intervention and control groups at 6 months [24], as well as at 6 and 12 months [26]. However, the use of the chatbot had no statistically significant effect between groups at 12 months [24]. The results of Chinkam’s study [20] are not included in Table 2, as it is a qualitative study with a small sample size (12 women). The findings of this study showed that women with previous cesarean sections and antenatal care providers viewed positively the use of an Embodied Conversational Agent (ECA) to support shared decision-making about the mode of delivery after a previous cesarean section. Participants commented that although the ECA might seem somewhat “robotic”, it could provide easy access to information for patients and complement consultation with care providers. It was suggested that improvements be made to the visual appeal of the ECA, and that the role and timing of decision tools using ECA technology be clarified to improve the shared decision-making process [20].

### 3.2. Other Outcomes Measured

We carried out an additional analysis by secondary outcomes, which we categorized into three categories (Antecedents, Healthy Behaviors, and Health status or health services utilization). By antecedents, we mean outcomes related to usability (usefulness, ease of learning, feasibility, acceptability, engagement, trust, and satisfaction) as well as outcomes related to knowledge, attitude, and behavioral intention. As presented in Table 3, some authors evaluated the experience of using the technology [29] and usability [23,26,29], while others assessed participant engagement with the chatbot by measuring the number of interactions with the chatbot, the number of logins [29], and the duration of each session [29]. Other outcomes documented were the impact of chatbot interactions on improving levels of nutrition knowledge and adoption of dietary habits [23], knowledge related to food insecurity, and attitudes towards breastfeeding, confidence in breastfeeding, and intention to breastfeed exclusively [22]. Chung et al. [21] focused on the ease of learning with the chatbot. For their part, Jack et al. [26] and Gardiner et al. [24] were interested in demonstrating how the chatbot can help its users navigate the behavior-change process through the stages of change and achieving a sense of self-efficacy [22,28]. Other outcomes assessed included feasibility and acceptability [20], trust [26], and satisfaction [19,21,22]. Details are provided in Table 3. Based on the results of our review, user experience and the satisfaction of new mothers with the chatbot reports showed that women’s level of agreement with the simplicity, good quality of information, clarity of content, usefulness, and overall satisfaction with the chatbot, was over 90% [18]. The Gabby system was also reported as usable for delivering lifestyle modifications and as easy to use and navigate [23].

Participants engaging with the chatbot demonstrated higher satisfaction levels in comparison to those utilizing patient education sheets, and they also expressed a willingness to recommend the system to others. Nguyen et al. [33] and Suharwardy et al. [34] pointed in the same direction by highlighting the high user satisfaction and usability of health chatbots, particularly among postpartum women seeking breastfeeding support. Despite some technical issues, most users expressed overall satisfaction with the platform’s usability and reported positive experiences with the chatbot interface. These findings underscore the importance of chatbots as an accessible, user-friendly resource for maternal health support, and offer promising potential for meeting user needs in this specific population.

### 3.3. Quality Assessment

We used the Mixed Methods Appraisal Tool (MMAT) to assess the quality of all included studies [31]. After this analysis, we found that four of the six included RCTs were of very good to excellent quality (4 or 5 stars). The other RCTs were of average quality (from 2 to 3 stars). The most common methodological limitations for RCTs were: (1) lack of certainty that participants would adhere to the interventions, or outcome data are not complete, or information about the blinding of evaluators is not provided, or there was no blinding.

The three mixed-method studies were from average (2 stars) to very good (4 stars) quality, and the quality of the descriptive observational studies was average (2 stars). The most common methodological limitations were: (1) divergences and inconsistencies between quantitative and qualitative results that are not adequately addressed; (2) lack of adherence to the quality criteria of each tradition of the methods involved; (3) lack of adequate rationale for using a mixed methods design to address the research question. The only qualitative study included in the review was of excellent quality (5 stars). (see Table 4).

## 4. Discussion

### 4.1. Main Findings

In the rapidly evolving landscape of digital health interventions, chatbots have emerged as promising tools for supporting women’s health. This systematic review aimed to identify and evaluate studies on chatbots designed to support women and expectant parents throughout the reproductive journey, from preconception to 12 months postpartum. Our review revealed the efficacy of chatbots in delivering perinatal care and promoting healthy lifestyles. Studies consistently demonstrated the feasibility of providing advice on physical activity, nutrition, mindfulness, and stress management through user-friendly chatbots. These digital tools, developed using text-mining techniques and contextual usability testing, proved particularly valuable in supporting women’s health across diverse urban settings.

The effectiveness of chatbots aligns with previous findings on mobile phone-based interventions during pregnancy [35]. Both approaches have shown positive impacts on maternal behaviors, contributing to improved maternal- and fetal health outcomes. Specifically, these digital interventions have been associated with increased self-reported physical activity, higher rates of smoking cessation, improved dietary habits including increased fruit, vegetable, and folic acid intake, and reduced alcohol consumption among pregnant women.

The majority of studies in our review reported high feasibility and user acceptance of chatbot interventions which is consistent with the literature. These digital tools have been successfully implemented across various health domains, including mental health support for young adults [36], HIV testing and pre-exposure prophylaxis promotion [37], and COVID-19 guidance for pregnant and breastfeeding women [38]. Notably, users expressed high satisfaction with both web-based and app-based chatbots, underscoring their versatility and broad appeal [39].

Our findings extend beyond chatbots to encompass broader internet-based prenatal interventions. These digital tools have demonstrated positive impacts on various aspects of maternal health, including satisfaction, parent–child bonding, breastfeeding efficacy, social support, and overall quality of life [40,41]. While these results suggest that chatbot interventions could be an effective strategy for supporting perinatal women’s health and well-being, we caution that careful consideration is needed when addressing anxiety in this population.

Recent research on perinatal women’s engagement with digital emotional well-being interventions [42] has highlighted the critical role of usability. Our review corroborates these findings, emphasizing the importance of user experience and ease of interaction in digital health tools. By prioritizing usability as a primary outcome, researchers and developers can create more effective and engaging interventions that better address the unique needs of women during the perinatal period.

Our review underscores the potential of chatbots as effective tools for promoting healthy behaviors and engaging women in managing their well-being. The implementation of chatbots has shown a significant impact on user engagement, particularly in reducing stress-related alcohol consumption and increasing daily fruit intake. Users have reported a greater utilization of stress management techniques, with many acknowledging that they have adopted the chatbot’s suggestions to help improve their stress levels. This indicates that chatbots not only provide valuable information but also encourage positive behavioral changes, making them a promising resource for enhancing health outcomes among women in urban settings.

These findings are further corroborated and expanded upon by a recent systematic review and meta-analysis of AI chatbot interventions in women’s health [41,43]. This comprehensive analysis has illuminated the significant potential of these technologies to enhance healthcare outcomes across a broader spectrum of women’s health issues. Chatbots have shown considerable promise in delivering health education, supporting mental health, managing chronic diseases, and providing targeted interventions for reproductive health and prenatal education. Notably, these interventions have been effective in improving both physical and mental health outcomes, particularly in reducing anxiety, underscoring the value of integrating AI chatbots into healthcare strategies for women.

The importance of leveraging digital technologies for preconceptional care was further emphasized by studies highlighting the role of chatbots in providing essential preconception information. These digital tools offer comprehensive support throughout the reproductive journey, empowering users to make informed health decisions and encouraging proactive health behaviors. Several studies in our review addressed preconception care risks both directly and indirectly. For instance, the Gabby system demonstrated effectiveness in delivering healthy lifestyle recommendations and addressing preconceptional risks among diverse populations, including African American and Black women [23,24,25]. Other studies focused on developing chatbots for perinatal care and parental support [21,29], indirectly contributing to preconceptional care through the promotion of maternal and infant health. For example, “Dina” a chatbot developed to inform and empower pregnant women with gestational diabetes mellitus promotes stable blood glucose and thereby prevent the development of adverse outcomes for the mother and the fetus [44]. In the same vein, “Wysa” is an AI-based emotionally intelligent mobile app aiming to build mental resilience and promote mental well-being in women with a self-reported maternal event, and its evaluation showed significant reductions in depressive symptoms [43].

However, it is important to recognize that equitable access to digital technologies is not guaranteed for all populations [45]. Disparities related to digital literacy, access to connected devices, and high-quality internet connections can limit adoption. These challenges are particularly pronounced in low-resource settings or among vulnerable groups such as migrants and refugees, or neurodiverse individuals [46,47,48]. Future research is needed to explore these issues and ensure that digital solutions are accessible and inclusive for all users.

In conclusion, our review reveals the multifaceted benefits of chatbots in improving health outcomes and enhancing user engagement across various stages of reproductive and maternal health. From preconception care to postpartum support and breastfeeding guidance, chatbots serve as valuable tools for disseminating knowledge and promoting proactive health behaviors. Their ability to provide anonymous, non-judgmental interactions makes them particularly effective in addressing sensitive health issues. As digital health continues to evolve, chatbots represent a promising avenue for delivering personalized, accessible, and effective support to women throughout their reproductive journey.

### 4.2. Strengths

This systematic review is underpinned by several methodological strengths that enhance its reliability and comprehensiveness. To ensure transparency and reproducibility, we pre-registered the study protocol with the International Prospective Register of Systematic Reviews (PROSPERO) and adhered strictly to the PRISMA guidelines throughout the review process, minimizing potential bias in our findings.

Our search strategy, developed and implemented by an experienced librarian, was both robust and comprehensive. We conducted searches in six databases, supplemented by hand searches to identify additional relevant studies. Notably, our literature search included five languages (English, French, Spanish, Portuguese, and Italian), allowing for a broad and inclusive review of the published literature on the topic.

To further reduce the risk of bias, two independent reviewers performed study selection and data extraction, with a consensus process for conflict resolution. This approach ensured agreement on the included studies and increased the reliability of our data synthesis. We used the Mixed Methods Appraisal Tool (MMAT) to critically appraise the methodological quality of the included studies, providing a standardized assessment of study rigor across our sample.

A key strength of our review is its comprehensive scope, covering the entire perinatal period from preconception through pregnancy and up to 12 months postpartum. This broad temporal range provides a holistic portrait of chatbot use across critical periods of maternal and infant life, allowing for a comprehensive understanding of their use and impact.

Together, these methodological strengths enhance the validity and reliability of our findings and provide a solid foundation for understanding the current state of chatbot use in perinatal care. By adhering to these rigorous standards, we aim to provide a trustworthy and comprehensive synthesis of the available evidence that can inform future research, clinical practice, and policy decisions in the area of digital health interventions for maternal and infant care.

### 4.3. Limitations

Despite our rigorous methodology, this systematic review has some limitations that should be considered when interpreting its results. The primary limitation was the limited number and heterogeneity of the included studies, which precluded the ability to perform a meta-analysis. This limitation hinders our ability to quantify the effect size of chatbot interventions on specific outcomes in perinatal care.

The paucity of qualitative studies in our sample, with only one such study included, limits our ability to provide a more nuanced, in-depth synthesis of user experiences and perspectives on chatbot interventions. This gap in qualitative data highlights the need for more diverse research approaches in this area.

Although our initial protocol included plans to search the grey literature, we ultimately decided to focus solely on peer-reviewed publications. This decision was made due to time constraints and the desire to ensure a high level of scientific rigor in our included studies. While this approach may have resulted in the exclusion of some relevant unpublished data, it allowed us to focus on evidence that has undergone rigorous peer review. We acknowledge that this departure from our original protocol may limit the comprehensiveness of our findings. Future reviews on this topic may benefit from including grey literature to capture a broader range of evidence on chatbot interventions in perinatal care.

Another limitation is the lack of data collection on the source of funding (private versus government) of the included studies. This information could provide important context for understanding the goals and focus of different interventions. For example, funding sources may influence whether an intervention targets a specific phase of the perinatal period (e.g., pregnancy) rather than the entire continuum, due to budgetary constraints or alignment with specific program goals.

In addition, our review did not include an analysis stratified by country income level. Such an analysis could have provided insights into how economic factors may influence the outcomes of maternal and child health interventions using chatbots, potentially revealing important differences or trends across economic contexts.

These limitations highlight areas for improvement in future research and reviews in this area. They underscore the need for more diverse and comprehensive studies of chatbot interventions in perinatal care, including more qualitative research, consideration of funding sources, and analysis of economic factors that may influence intervention outcomes.

### 4.4. Future Research Prospects

This systematic review highlights several key areas for future research in chatbot interventions for perinatal care. There is a notable lack of studies that include male partners and both parents in perinatal health interventions. Given the positive impact of male support on maternal and newborn health, it is crucial to develop chatbot interventions that engage fathers and assess their influence on health outcomes.

Additionally, the variability in defining the perinatal period calls for a standardized definition to enhance consistency across research. This will help clarify how different definitions affect intervention design and outcomes.

The preconception period also requires more attention, as it significantly impacts fetal development. Future studies should focus on designing chatbot interventions for this phase and exploring their long-term effects on maternal and child health.

Longitudinal research is essential to understand the sustained impacts of chatbot interventions, tracking participants from preconception through to postpartum. Finally, culturally adapting chatbot interventions for diverse populations will ensure their effectiveness across various contexts.

The chatbots included in our review are primarily second-generation systems, which predate the integration of large language models (LLMs). These earlier systems represent a significant shift from first-generation chatbots, limited to predetermined question-and-answer scripts. Second-generation chatbots incorporate more advanced rule-based logic and can simulate more dynamic interactions, but their capabilities are still constrained compared to recent LLM-powered systems. The latest generation of conversational agents powered by LLMs—such as ChatGPT—have revolutionized human–computer interactions by enabling more realistic and context-aware dialogue [49].

Therefore, our review serves as a critical baseline assessment of these second-generation chatbots, allowing for the identification of their limitations and potential. The rapid progress in perinatal care conversational agents calls for future research to evaluate the impact of advanced AI technologies on maternal and infant health outcomes. Additionally, studies should explore how LLM-powered tools affect user engagement, access to care, and digital inclusion across diverse populations, especially considering disparities in healthcare access [50,51,52].

## 5. Conclusions

Research on interventions using digital technologies is booming, but the use of interactive conversational agents in the perinatal period is still in its infancy, with a limited number of studies that are highly heterogeneous. Our analysis shows that digital interventions using interactive conversational agents have a positive impact on several aspects of perinatal health, including knowledge, behaviors, attitudes, and the use of health services. These interventions appear to be more effective than traditional methods, highlighting the transformative potential of chatbots in perinatal care.

As we look to the future, chatbots represent a paradigm shift in how we deliver perinatal health support. Their ability to provide personalized, accessible, and timely information has the potential to revolutionize prenatal education, increase maternal and paternal engagement in health behaviors, and ultimately improve outcomes for mothers, fathers, and infants. By providing continuous, nonjudgmental support, chatbots can address critical gaps in care, particularly in underserved areas or for sensitive topics that individuals may be reluctant to discuss with healthcare providers.

However, realizing this potential requires overcoming current challenges. Innovative strategies are needed to increase engagement, reduce attrition, and engage partners at all stages of the perinatal period. In addition, integrating behavior-change theories and techniques into chatbot design is critical to optimizing their effectiveness, particularly in preconception interventions.

As we continue to refine and expand the use of chatbots in perinatal care, we are on the cusp of a digital revolution in maternal and child health. By harnessing the power of artificial intelligence and personalized digital support, we have the opportunity to create a future where every parent and child benefits from accessible, high-quality perinatal care, ultimately leading to healthier families and communities worldwide.

## Figures and Tables

**Table 1:** Characteristics of the included studies.

Studies	Country	Topics Covered	Study Population	Study Participants	Name of Chatbot	Functionalities	Study Design
Barreto, 2021 [18]	Brazil	Child health promotion	Mothers of new-borns	142	GISSA Mother-Baby	Text	Cross-sectional research with mixed study
Bickmore, 2020 [19]	USA	Preconception care risks	Female, Black, or African American aged 18–34 years, not pregnant	262	Gabby	Text Voice Avatar	Experimental study: randomized clinical trial
Chinkam, 2021 [20]	USA	Mode of birth after cesarean	Women with a previous cesarean and their prenatal providers	20	–	Audio Text Voice Avatar	Qualitative study
Chung, 2021 [21]	Republic of Korea	Obstetric and mental health care	Men aged between 38 and 40 years and women aged from 27 to 43 years	15	Dr. Joy	Text Voice	Observational study: descriptive study
Edwards, 2013 [22]	USA	Intent to breastfeed, attitudes towards breastfeeding, breastfeeding self-efficacy, exclusive breastfeeding expectation	Primipara, pregnant in the third trimester with one fetus, 18 years of age or older	15	Tanya	Text Avatar	Experimental study: randomized clinical trial
Gardiner, 2017 [23]	USA	Lifestyle changes	Women, 18–50 years	57	Gabby	Audio Text Avatar	Mixed study
Gardiner, 2021 [24]	USA	Preconception health risks	African American or Black women, ages 18–34 years	229	Gabby	Text Avatar	Experimental study: randomized clinical trial
Jack, 2015 [25]	USA	Preconception health risks	African American or Black women, 18–34 years of age	77	Gabby	Audio Text Avatar	Experimental study: randomized clinical trial
Jack, 2020 [26]	USA	Preconception related risks	African American or Black women	528	Gabby	Text Voice Avatar	Experimental study: randomized clinical trial
Maeda, 2020 [27]	Japan	Fertility and preconception health	Women aged between 20 and 34 years	927	–	Text	Experimental study: randomized clinical trial
Montenegro, 2022 [28]	Brazil	Preconception health	Pregnant women in the prenatal or postnatal stages	20	Maria	Text	Mixed study
Wong, 2021 [29]	Singapore	Stress, sleep, infant feeding	Parents (women) aged ≥21 years	26	ClaimIt	Text	Observational descriptive study: multi-stage

**Table 2:** Primary outcomes.

Studies		Usability/ Feasibility	Preconception Risks	Knowledge	Breastfeeding
Barreto, 2021 [18]		∗	–	–	–
Bickmore, 2020 [19]		∗	–	–	–
Chung, 2021 [21]		√	–	–	–
Edwards, 2013 [22]		–	–	–	√
Gardiner, 2017 [23]		√	–	√	–
Gardiner, 2021 [24]	At 6 months	–	√	–	–
	At 12 months	–	0	–	–
Jack, 2015 [25]	At 6 months	–	√	–	–
Jack, 2020 [26]	At 6 months	–	√	–	–
	At 12 months	–	√	–	–
Maeda, 2020 [27]	Intervention vs. control 1 (no chatbot)	–	–	√	–
	Intervention vs. control 2 (PDF document on irrelevant topic)	–	–	–	–
Montenegro, 2022 [28]		∗	–	–	–
Wong, 2021 [29]		∗	–	–	–

**Legend:** √: Significant positive effect; 0: not statistically significant; ∗: positive correlation with no significant effect; –: not evaluated.

**Table 3:** Other Outcomes.

Studies	Antecedents	Healthy Behaviors	Health Status or Health Services Utilization
Barreto, 2021 [18]	N/A	–	–
Bickmore, 2020 [19]	▪ Chatbot usability at 6 and 12 months: 62.9% and 67.9% (non-statistically significant). ▪ Satisfaction with chatbot at 6 and 12 months: 80.0% and 85.7% (non-statistically significant).	–	–
Chinkam, 2021 [20]	▪ Feasibility/Acceptability: The chatbot could support provider and patient discussions and offer programmed consistency in preparatory information.	–	–
Chung, 2021 [21]	▪ Usefulness: Less than half (M (SD) = 4.87 (1.11)) of the participants find the chatbot useful. ▪ Ease of use: More than half of the participants (M (SD) = 5.34 (0.73)) find the chatbot easy to use. ▪ Ease of learning: More than half of the participants (M (SD) = 6.35 (0.71)) find the chatbot easy to learn. ▪ Satisfaction: Less than half of the participants (M (SD) = 4.90 (1.26)) are satisfied with the chatbot.	–	–
Edwards, 2013 [22]	▪ Breastfeeding/Self-Efficacy: Higher for intervention group (58.7) than control group (54.1); *p* = 0.35. ▪ Satisfaction with the chatbot: 5.7/7-point scale for both the prenatal visit and perinatal visit (SD = 1.38 and 1.37, respectively). ▪ Confidence with the chatbot: 5.9/6.7-point scale for the prenatal visit (SD = 1.1) and perinatal visit 6.7 (SD = 0.5). ▪ Attitudes toward breastfeeding: No significant differences between groups.	–	–
Gardiner, 2017 [23]	▪ No significant difference in food knowledge, food insecurity, and breakfast consumption between groups (*p* = 0.15, *p* = 0.99, and *p* = 0.11, respectively).	▪ Physical activity: 52% of women utilized suggestions from Gabby to increase physical activity compared to 49% of women who utilized information sheet. This difference is not statistically significant	–
Gardiner, 2020 [24]	▪ Total usage: 198 of the 240 women in the IG interacted at least once with the entire Gabby system. ▪ After 12 months: - Median number of logins = 6. - Median duration per session = 13.7 min. ▪ Stage of change (food choices subdomain): - At 6 months: IG versus CG = 62.76% versus 49.17%, *p* = 0.165. - At 12 months: IG versus CG = 73.33% versus 62.38%, *p* = 0.401.	–	–
Jack, 2020 [26]	▪ Progressing forward on the stage of change scale: - At 6 months: IG versus CG = 42.1% (SD = 26.2) versus 35.5% (SD = 23.2); *p* = 0.00012. - At 12 months: IG versus CG = 43.7% (SD = 27.1) versus 40.2% (SD = 5.4); *p* = 0.071. ▪ Regressing backward on the stage of change scale: - At 6 months: IG versus CG = 18.9% (SD = 22.1) versus 22.3% (SD = 22.3); *p* = 0.01. - At 12 months: IG versus CG = 18.1% (SD = 20.4) versus 20.4% (21.4); *p* = 0.03. ▪ Use of the system Gabby: 76/118 (64%) of respondents rated it easy to use. ▪ Trust: 75/110 (68%) respondents trusted Gabby (much or very much).	–	▪ Clinical visits at 12 months: IG versus CG (587 vs. 812; *p* = 0.02).
Jack, 2015 [25]	▪ Average session lasted: 18.6 (SD = 12.1) minutes. ▪ Average interaction time with Gabby during the study: 63.7 (SD = 70.4, range 2.8–286) minutes per woman.	–	–
Maeda, 2020 [27]	–	–	▪ Post-test state anxiety scores on the STAI (mean ± SD): - IG: (43.2 ± 9.5), *p* < 0.001. - CG 1: (47.5 ± 9.5) < CG 2: (46.2 ± 9.0), *p* < 0.001.
Montenegro, 2022 [28]	▪ Self-efficacy: - Mean according to feeling intimidated by using the chatbot for pregnant women: (mean = 4.00). - Mean for agreement on facilitating conditions: (avg = 3.07).	–	–
Wong, 2021 [29]	▪ Preterm and term groups: Scored between «neutral» and «satisfied» with the chatbot, respectively, 3.62 (SD = 0.96) and 4.0, (SD = 0.82). ▪ Length of interactions: - Preterm group: Interaction was between «long» to «neutral» (mean = 2.92, SD = 1.19). - Term group: Interaction was between «manageable» and «easily manageable» (mean = 4.31, SD = 0.48). ▪ Experience of technical issues when using the chatbot: 46% (6/13) of the preterm parents and 23% (3/13) of the term parents.	–	–

**Legend:** IG: Intervention group; CG: control group.

**Table 4:** Quality assessment of included studies based on the Mixed Methods Appraisal Tool.

Authors	Study Design	Quantitative RCT	Quantitative Descriptive	Mixed Methods	Qualitative
Barreto, 2021 [18]	Cross-sectional research with mixed study			(4/5) ****	
Bickmore, 2020 [19]	Experimental study: randomized clinical trial	(3/5) ***			
Chinkam, 2021 [20]	Qualitative study				(5/5) *****
Chung, 2021 [21]	Observational study: descriptive study		(2/5) **		
Edwards, 2013 [22]	Experimental study: randomized clinical trial	(4/5) ****			
Gardiner, 2017 [23]	Mixed study	(2/5) **			
Gardiner, 2020 [24]	Experimental study: randomized clinical trial	(2/5) **			
Jack, 2015 [25]	Experimental study: randomized clinical trial	(4/5) ****			
Jack, 2020 [26]	Experimental study: randomized clinical trial	(5/5) *****			
Maeda, 2020 [27]	Experimental study: randomized clinical trial	(5/5) *****			
Montenegro, 2022 [28]	Mixed study	(3/5) ***			
Wong, 2021 [29]	Observational descriptive study: multi-stage		(2/5) **		

**Legend:** **: Two stars; ***: Three stars; ****: Four stars; *****: Five stars.

## Data Availability

The extracted data have been transposed into the results tables and the search strategy is provided in Appendix A. The data extraction grid can be shared upon reasonable request to the corresponding author. The corresponding author can be contacted for further information.

## Appendix A

**Databases Search Strategy**

Medline (OVID)

Date of the search: 09 November 2022

**Database Limit: Limit Results to Publications Date Between 23 July 2021 and 9 November 2022**	**Search Strategy**	**Results**
1	(“chat bot?” or chatterbot? or chatbot? or medbot? or “chatter bot?” or smart bot? or smartbot?).ti,ab,kw,kf	574
2	(Conversational adj2 (host or coach or avatar or advisor or “Artificial Intelligence” or interface or avatar or agent? or system or computer or humanoid or character or bot? or AI)).ti,ab,kw,kf	475
3	((virtual or intelligent or chat or computer or AI or “artificial intelligence” or relational or embodied) adj2 agent?).ti,ab,kw,kf	1022
4	((Conversational OR Virtual OR Voice OR “Artificial Intelligence” OR Digital) adj2 assistan*).ti,ab,kw,kf	1528
5	1 or 2 or 3 or 4	3186
6	Exp perinatal Care/OR Perinatology/OR exp “Infant, Newborn”/ OR Pregnant Women/OR Pregnancy/OR Obstetrics/	1451583
7	newborn?.ti,ab,kw,kf OR Neonat*.ti,ab,kw,kf OR Pregnan*.ti,ab,kw,kf OR Perinat*.ti,ab,kw,kf OR Matern*.ti,ab,kw,kf OR Postpartum.ti,ab,kw,kf OR Postnatal.ti,ab,kw,kf OR Childbirth?.ti,ab,kw,kf OR Obstetric?.ti,ab,kw,kf OR “post natal”.ti,ab,kw,kf	1268687
8	6 or 7	1907674
9	5 AND 8	77
10	limit 9 to ed = 20210723-20221109	8

Embase (Embase.com)

(accessed on 09 November 2022).

Database limit: limit results to publications date between 23 July 2021 and 09 November 2022

**#**	**Search Strategy**	**Results**
1	(“chat bot$” OR chatterbot$ OR chatbot$ OR medbot$ OR “chatter bot$” OR “smart bot$” OR smartbot$):ti,ab,kw	576
2	(Conversational NEAR/2 (host OR coach OR avatar OR advisor OR “Artificial Intelligence” OR interface OR avatar OR agent$ OR system OR computer OR humanoid OR character OR bot$ OR AI)):ti,ab,kw	419
3	((virtual OR intelligent OR chat OR computer OR AI OR “artificial intelligence” OR relational OR embodied) NEAR/2 agent$):ti,ab,kw	1054
4	((Conversational OR Virtual OR Voice OR “Artificial Intelligence” OR Digital) NEAR/2 assistan*):ti,ab,kw	1841
5	#1 OR #2 OR #3 OR #4	3566
6	‘perinatal care’/exp OR ‘newborn’/de OR ‘pregnant woman’/de OR ‘obstetric procedure’/de OR ‘postnatal care’/exp OR ‘pregnancy’/de OR ‘hildbirth’/de OR ‘perinatology’/de OR ‘puerperium’/de	1,481,462
7	newborn$:ti,ab,kw OR Neonat*:ti,ab,kw OR Pregnan*:ti,ab,kw OR Perinat*:ti,ab,kw OR Matern*:ti,ab,kw OR Postpartum:ti,ab,kw OR Postnatal:ti,ab,kw OR Childbirth$:ti,ab,kw OR Obstetric$:ti,ab,kw OR “post natal”:ti,ab,kw OR puerperium:ti,ab,kw	1,633,076
8	#6 OR #7	2,168,193
9	#5 AND #8	98
10	#9 AND [23-07-2021]/sd	16

CINAHL

Date of the search: 09 November 2022

Database limit: limit results to publications date between 23 July 2021 and 09 November 2022

**#**	**Search Strategy**	**Results**
1	TI (“chat bot?” OR chatterbot? OR chatbot? OR medbot? OR “chatter bot?” OR smart bot? OR smartbot?) OR AB (“chat bot?” OR chatterbot? OR chatbot? OR medbot? OR “chatter bot?” OR smart bot? OR smartbot?)	306
2	TI (Conversational N2 (host OR coach OR avatar OR advisor OR “Artificial Intelligence” OR interface OR avatar OR agent? OR system OR computer OR humanoid OR character OR bot? OR AI)) OR AB (Conversational N2 (host OR coach OR avatar OR advisor OR “Artificial Intelligence” OR interface OR avatar OR agent? OR system OR computer OR humanoid OR character OR bot? OR AI))	194
3	TI ((virtual OR intelligent OR chat OR computer OR AI OR “artificial intelligence” OR relational OR embodied) N2 agent?) OR AB ((virtual OR intelligent OR chat OR computer OR AI OR “artificial intelligence” OR relational OR embodied) N2 agent?)	306
4	TI ((Conversational OR Virtual OR Voice OR “Artificial Intelligence” OR Digital) N2 assistan*) OR AB ((Conversational OR Virtual OR Voice OR “Artificial Intelligence” OR Digital) N2 assistan*)	981
5	S1 OR S2 OR S3 OR S4	1637
6	MH “Perinatal Care” OR MH “Maternal-Child Care” OR MH Perinatology OR MH “Expectant Mothers” OR MH “Infant, Newborn+” OR MH “Postnatal Care” OR MH “Prepregnancy Care” OR MH “Obstetric Care” OR MH “Childbirth” OR MH” Postnatal Period” OR MH Puerperium OR MH Pregnancy	367,369
7	TI newborn# OR AB newborn# OR TI Neonat* OR AB Neonat* OR TI Pregnan* OR AB Pregnan* OR TI Perinat* OR AB Perinat* OR TI Matern* OR AB Matern* TI newborn# OR AB newborn# OR TI Neonat* OR AB Neonat* OR TI Pregnan* OR AB Pregnan* OR TI Perinat* OR AB Perinat* OR TI Matern* OR AB Matern*	307,158
8	S6 OR S7	471,873
9	S5 AND S8	36
10	S9 AND DT 20210723-20221109	2

Web of Science

Date of the search: 09 November 2022

Database limit: database limit publications date between 23 July 2021and 09 November 2022 has been applied

**#**	**Search Strategy**	**Results**
1	TS = (“chat bot$” OR chatterbot$ OR chatbot$ OR medbot$ OR “chatter bot$” OR smart bot$ OR smartbot$)	7667
2	TS = (Conversational NEAR/2 (host OR coach OR avatar OR advisor OR “Artificial Intelligence” OR interface OR avatar OR agent$ OR system OR computer OR humanoid OR character OR bot$ OR AI))	940
3	TS = ((virtual OR intelligent OR chat OR computer OR AI OR “artificial intelligence” OR relational OR embodied) NEAR/2 agent$)	1389
4	TS = ((Conversational OR Virtual OR Voice OR “Artificial Intelligence” OR Digital) NEAR/2 assistan*)	949
5	#1 OR #2 OR #3 OR #4	10,146
6	TS = (newborn$ OR Neonat* OR Pregnan* OR Perinat* OR Matern* OR Postpartum OR Postnatal OR Childbirth$ OR Obstetric$ OR “post natal” OR puerperium)	91,419
7	#5 AND #6	39

Inspec (Engineering Village)

Date of the search: 09 November 2022

Database limit: database limit publications date between 23 July 2021 and 09 November 2022 has been applied

**#**	**Search Strategy**	**Results**
1	chatbots WN CV	527
2	“chat bot*” WN TI OR chatterbot* WN TI OR chatbot* WN TI OR medbot* WN TI OR “chatter bot*” WN TI OR smart bot* WN TI OR smartbot* WN TI OR “chat bot*” WN AU OR chatterbot* WN AU OR chatbot* WN AU OR medbot* WN AU OR “chatter bot*” WN AU OR smart bot* WN AU OR smartbot* WN AU	591
3	(Conversational NEAR/2 host) WN TI OR (Conversational NEAR/2 coach) WN TI OR (Conversational NEAR/2 avatar) WN TI OR (Conversational NEAR/2 advisor) WN TI OR (Conversational NEAR/2 Intelligence) WN TI OR (Conversational NEAR/2 interface) WN TI OR (Conversational NEAR/2 avatar) WN TI OR (Conversational NEAR/2 agent*) WN TI OR (Conversational NEAR/2 system) WN TI OR (Conversational NEAR/2 computer) WN TI OR (Conversational NEAR/2 humanoid) WN TI OR (Conversational NEAR/2 character) WN TI OR (Conversational NEAR/2 bot*) WN TI OR (Conversational NEAR/2 AI) WN TI OR (Conversational NEAR/2 host) WN AU OR (Conversational NEAR/2 coach) WN AU OR (Conversational NEAR/2 avatar) WN AU OR (Conversational NEAR/2 advisor) WN AU OR (Conversational NEAR/2 Intelligence) WN AU OR (Conversational NEAR/2 interface) WN AU OR (Conversational NEAR/2 avatar) WN AU OR (Conversational NEAR/2 agent*) WN AU OR (Conversational NEAR/2 system) WN AU OR (Conversational NEAR/2 computer) WN AU OR (Conversational NEAR/2 humanoid) WN AU OR (Conversational NEAR/2 character) WN AU OR (Conversational NEAR/2 bot*) WN AU OR (Conversational NEAR/2 AI) WN AU	224
4	(virtual NEAR/2 agent) WN TI OR (intelligent NEAR/2 agent) WN TI OR (chat NEAR/2 agent) WN TI OR (computer NEAR/2 agent) WN TI OR (AI NEAR/2 agent) WN TI OR (“artificial intelligence” NEAR/2 agent) WN TI OR (relational NEAR/2 agent) WN TI OR (embodied NEAR/2 agent) WN TI OR (virtual NEAR/2 agent) WN AU OR (intelligent NEAR/2 agent) WN AU OR (chat NEAR/2 agent) WN AU OR (computer NEAR/2 agent) WN AU OR (AI NEAR/2 agent) WN AU OR (“artificial intelligence” NEAR/2 agent) WN AU OR (relational NEAR/2 agent) WN AU OR (embodied NEAR/2 agent) WN AU	115
5	(Conversational NEAR/2 assistan*) WN TI OR (Virtual NEAR/2 assistan*) WN TI OR (Voice NEAR/2 assistan*) WN TI OR (“Artificial Intelligence” NEAR/2 assistan*) WN TI OR (Digital NEAR/2 assistan*) WN TI OR (Conversational NEAR/2 assistan*) WN AB OR (Virtual NEAR/2 assistan*) WN TI OR (Voice NEAR/2 assistan*) WN AB OR (“Artificial Intelligence” NEAR/2 assistan*) WN AB OR (Digital NEAR/2 assistan*) WN AB	1402
6	#1 OR #2 OR #3 OR #4 OR #5	2386
7	newborn* WN TI OR newborn* WN AB OR Neonat* WN TI OR Neonat* WN AB OR Pregnan* WN TI OR Pregnan* WN AB OR Perinat* WN TI OR Perinat* WN AB OR Matern* WN TI OR Matern* WN AB OR Postpartum WN TI OR Postpartum WN AB OR Postnatal WN TI OR Postnatal WN AB OR Childbirth* WN TI OR Childbirth* WN AB OR Obstetric* WN TI OR Obstetric* WN AB OR “post natal” WN TI OR “post natal” WN AB OR puerperium WN TI OR puerperium WN AB	16,208
8	#6 AND #7	5

IEEE Xplore

Date of the search: 09 November 2022

Database limit: limit results to publications date between 23 July 2021 and 09 November 2022

**#**	**Search Strategy**	**Results**
1	“All Metadata”:chatbot AND (Pregnant OR pregnancy OR perinatal)	3

Google Scholar (https://harzing.com/resources/publish-or-perish)

(accessed on 9 November 2022).

Database limits: only up to the 20 first results per string have been considered; publications between 2021 and 2022 limit has been applied; citations and patents options have been removed

**#**	**Search**	**# Results Screened**
1	“Conversational agent” AND (Pregnant OR pregnancy OR perinatal)	20
2	Conversational AND assistant AND (Pregnant OR pregnancy OR perinatal)	20
3	chatbot AND (Pregnant OR pregnancy OR perinatal)	20
4	chatbots AND (Pregnant OR pregnancy OR perinatal)	20
	Total number of results	80
